# Development of a RPA-CRISPR/Cas12a based rapid visual detection assay for Porcine Parvovirus 7

**DOI:** 10.3389/fvets.2024.1440769

**Published:** 2024-09-09

**Authors:** Shubo Wen, Lemuge She, Sheng Dang, Ao Liao, Xiaorui Li, Shuai Zhang, Yang Song, Xiangyang Li, Jingbo Zhai

**Affiliations:** ^1^College of Animal Science and Technology, Inner Mongolia Minzu University, Tongliao, China; ^2^Brucellosis Prevention and Treatment Technology Research Center, Tongliao, China; ^3^Key Laboratory of Zoonose Prevention and Control at Universities of Inner Mongolia Autonomous Region, Tongliao, China; ^4^Guangzhou Yitun Pig Industry Co. Ltd., Guangzhou, China

**Keywords:** CRISPR/Cas12a, Porcine Parvovirus 7, detection, visual, PCR

## Abstract

**Introduction:**

Porcine Parvovirus (PPV) is a significant pathogen in the pig industry, with eight genotypes, including PPV7, identified since its emergence in 2016. Co-infections with viruses such as Porcine Circovirus 2 (PCV2) and Porcine Reproductive and Respiratory Syndrome Virus (PRRSV) pose serious risks to swine health. Thus, there is an urgent need for rapid, sensitive, and specific detection methods suitable for use in field settings or laboratories with limited resources.

**Methods:**

We developed a CRISPR/Cas12a-based assay combined with recombinase polymerase amplification (RPA) for the rapid detection of PPV7. Specific RPA primers and five CRISPR RNAs (crRNAs) were designed to target a highly conserved region within the NS1 gene of PPV7. Optimization of crRNA and single-stranded DNA (ssDNA) concentrations was performed to enhance the assay’s performance.

**Results:**

CrRNA optimization identified crRNA-05 as the optimal candidate for Cas12a-based detection of PPV7, as all synthesized crRNAs demonstrated similar performance. The optimal crRNA concentration was determined to be 200 nM, yielding consistent results across tested concentrations. For ssDNA optimization, the strongest fluorescence signal was achieved with 500 nM of the FAM-BHQ ssDNA receptor. The assay showed a minimal detection limit of 100copies/μl for PPV7, confirmed through fluorescence and lateral flow detection methods. Specificity testing indicated that only PPV7 DNA samples returned positive results, confirming the assay’s accuracy. In tests of 50 lung tissue samples from diseased pigs, the RPA-Cas12a assay identified 29 positive samples (58%), surpassing the 22 positive samples (44%) detected by conventional PCR. This highlights the RPA-Cas12a method’s enhanced detection capability and its potential utility in clinical surveillance and management of PPV7 in swine populations.

**Discussion:**

The RPA-Cas12a assay effectively detects PPV7 in clinical samples, enhancing disease surveillance and control in pigs. Its adaptability to resource-limited settings significantly improves PPV7 management and prevention strategies, thereby supporting the overall health and development of the pig industry.

## 1 Introduction

Porcine Parvoviruses (PPV), members of the family Parvovirinae, are small, nonenveloped, single-stranded DNA viruses with a genome size ranging from approximately 4 to 6.3 kb. To date, a total of 8 subtypes of PPVs (PPV1-PPV8) have been characterized. Taxonomically, PPV1 is classified within the genus Protoparvovirus, while the more recently identified genotypes (PPV2-PPV7) have been categorized into three distinct genera: Tetraparvovirus (PPV2-PPV3), Copiparvovirus (PPV4-PPV6), and Chapparvovirus (PPV7), based on the amino acid sequence similarity of the NS1 protein (1, 2). A new species, PPV8, was discovered in 2022 and is currently considered to belong to the same genus as the original PPV1 (Protoparvovirus). However, its taxonomic classification has not yet been officially determined (3). PPV1, first detected in Germany in 1965 (4, 5), was the only PPV genotype that has been determined to be associated with reproductive failure in pigs. As a recently discovered PPV, PPV7 was first identified in the USA in 2016 from rectal swab samples of healthy pigs using next-generation sequencing (2). Since then, PPV7 has been reported in various countries including Sweden (6), Poland (7, 8), Korea (9–11), China (12–17), Brazil (18) and Colombian (19), underscoring its global distribution and significance in the context of porcine health.

While the clinical manifestations resulting from PPV2 to PPV8 infections are not yet fully understood, PPV7 has been linked to certain interactions with other swine viruses. Studies have indicated that PPV7 can lead to increased PCV2 viremia in individual pigs (8) and elevate the viral nucleic acid levels of PCV3 in the serum of infected animals (16). This suggests that PPV7 may exacerbate the clinical symptoms associated with PCV2 or PCV3 infections (9). Furthermore, a study from Korea has reported an association between PPV7 and PRRSV infection (11). These findings highlight the potential impact of PPV7 on the severity and outcomes of co-infections with other swine viruses. Given the lack of effective vaccines or specific treatments for PPV7, there is a pressing need for the development of accurate and real-time diagnostic methods to facilitate the control and management of PPV7 spread and its potential interactions with other swine pathogens. Such diagnostic tools would be crucial for timely intervention and disease management in swine populations.

The clustered regularly interspaced short palindromic repeats (CRISPR) and CRISPR-associated (Cas) protein systems have been recognized for their ability to target and cleave specific nucleic acid sequences guided by CRISPR RNAs (crRNAs) in a process known as cis-cleavage (20, 21). In addition to this targeted cleavage, certain Cas proteins such as Cas12a, Cas12b, Cas13a, and Cas14 have demonstrated the capability for nonspecific trans-cleavage of non-target sequences following the recognition of a target DNA template (22–25). This property has led to the application of CRISPR-based detection methods in the identification of various nucleic acids, particularly those from pathogenic microorganisms.

However, the CRISPR/Cas12a or Cas13a system alone is supposed not sensitive enough to recognize low levels of nucleic acids, efforts have been made to enhance their detection capabilities. Integration of CRISPR/Cas12a or Cas13a with polymerase chain reaction (PCR) or isothermal amplification techniques such as recombinase polymerase amplification (RPA), loop-mediated isothermal amplification (LAMP), and cross-priming amplification has been pursued in recent years (23, 26–28). RPA, as a reliable nucleic acids detection method, could allow sensitive, specific and rapid isothermal DNA amplification at a temperature ranging from 37–42°C (29). The systems combined Cas12a or Cas13a with RPA and a fluorescence readout or lateral-flow strip with high sensitivities, specificities and convince have been proved to be applicable to pathogens detection (30, 31).

In this study, we intended to establish a feasible, specific, sensitive and reliable RPA-CRISPR/Cas12a based visual detection method for rapid detection of PPV7.

## 2 Materials and methods

### 2.1 Clinical samples and nucleic preparation

The viral genomic DNA of PPV2-PPV6, PCV2, PCV3, PRV, cDNA of PRRSV were stored in our laboratory. The lung tissues samples of sick pigs were received from different pig farms in eastern Inner Mongolia Autonomous Region and stored in our laboratory.

### 2.2 PPV7 target and primer design

**Figure 1:**
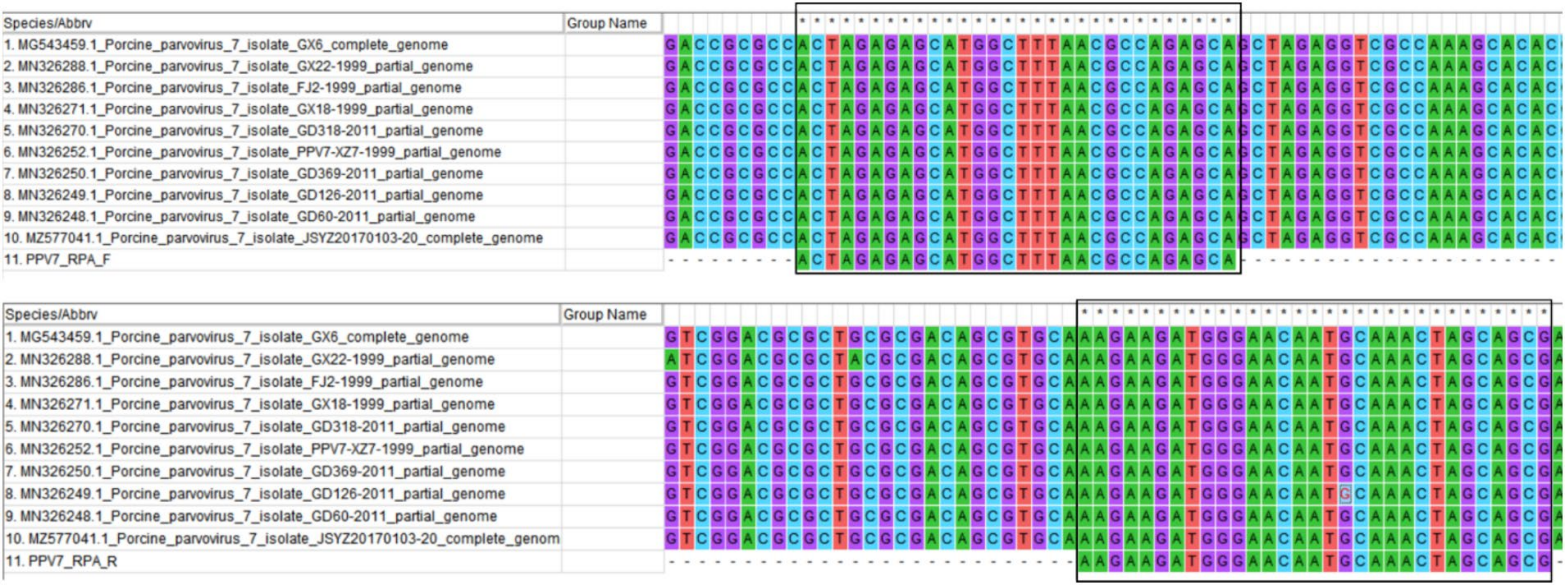
The primer pair designed for RPA amplification was algined with the NS1 gene of ten randomly selected PPV7 strains from GenBank using MEGA 7.0 software. The primer sequences are indicated with solid black boxes.

The NS1 gene sequences of PPV7 strains identified from different countries available in the GenBank database are highly conserved. A conservative region within the PPV7 NS1 gene was chosen as the target sequence, which was then synthesized and cloned into a pUC57 plasmid, designated as the pUC57-NS1 plasmid. RPA primers (RPA-F and RPA-R) were designed using Primer Premier 5.0 to target the pUC57-NS1 plasmid, following the principles of RPA primer design for amplification (32). The conservation of the primers was assessed by aligning them with the NS1 sequences of ten randomly selected PPV7 strains from GenBank (Figure 1). Additionally, the specificity of the RPA primers was evaluated using Primer-BLAST from NCBI. The crRNAs were designed to recognize specific regions within the PPV7 NS1 gene present in the RPA amplicon. Each crRNA binds to a 20-bp target sequence adjacent to a PAM site (TTTN or NAAA). Five crRNAs were designed based on the PPV7 NS1 gene sequence. Additionally, a pair of primers targeting the conserved NS1 region of PPV7 were designed for traditional PCR detection of PPV7 in clinical samples. All primers and crRNAs listed in Table 1 were synthesized by Sangon Biotech (Shanghai, China).

**Table 1:** The primers used in the PCR reaction and the CRISPR guide RNAs (crRNAs) in the Cas12a based reactions.

Name	Sequence (5′-3′)	Product (bp)
RPA-F	ACTAGAGAGCATGGCTTTAACGCCAGAGCA	304 bp
RPA-R	CGCTGCTAGTTTGCATTGTTCCCATCTTCTT	
crRNA-01	uaauuucuacuaaguguagauACGCCAGAGCAGCUAGAGGU	
crRNA-02	uaauuucuacuaaguguagauCUACUAUCGCAACUCAAGAC	
crRNA-03	uaauuucuacuaaguguagauUCUCUGCCACGAAGCACCAA	
crRNA-04	uaauuucuacuaaguguagauACCAGGCAGUGGUAGUGCAG	
crRNA-05	uaauuucuacuaaguguagauCAUUGUUCCCAUCUUCUCUG	
PCR-F	CTGAGCAACGGCGAAGGA	103 bp
PCR-R	CTGCCACGAAGCACCAAT	

### 2.3 RPA-Cas12a based assay

**Figure 2:**
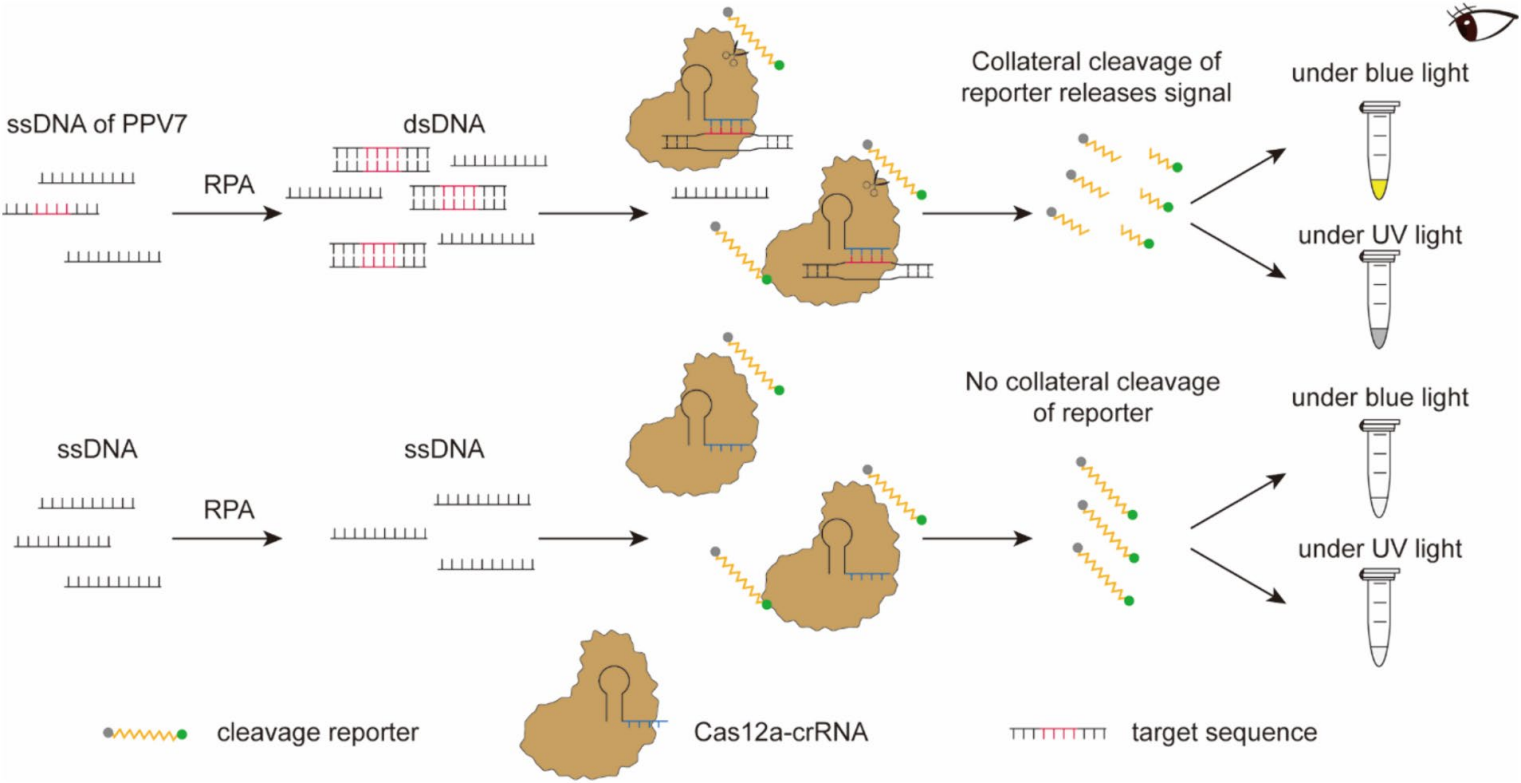
Schematic diagram of the RPA-CRISPR/Cas12a-based nucleic acid detection platform. Following amplification, the RPA product is employed for the detection assay using CRISPR/Cas12a. An ssDNA tagged with a quenched green fluorescent molecule is introduced for visual detection purposes. When the target molecule is present in the system, Cas12a cleaves the ssDNA, triggering a visible green fluorescence signal. In the absence of virus-derived dsDNA, the ssDNA remains uncleaved, resulting in no fluorescence signal generation. The detection outcomes can be directly observed by the naked eyes under blue light or UV light after an incubation period of approximately 15 min.

The RPA-CRISPR/Cas12a-based nucleic acid detection platform operates as follows (Figure 2). Following amplification, the RPA product is employed for the detection assay using CRISPR/Cas12a. An ssDNA tagged with a quenched green fluorescent molecule is introduced for visual detection purposes. When the target molecule is present in the system, Cas12a cleaves the ssDNA, triggering a visible green fluorescence signal. In the absence of virus-derived dsDNA, the ssDNA remains uncleaved, resulting in no fluorescence signal generation. The detection outcomes can be directly observed by the naked eyes under blue light or UV light after an incubation period of approximately 15 min.

**Figure 3:**
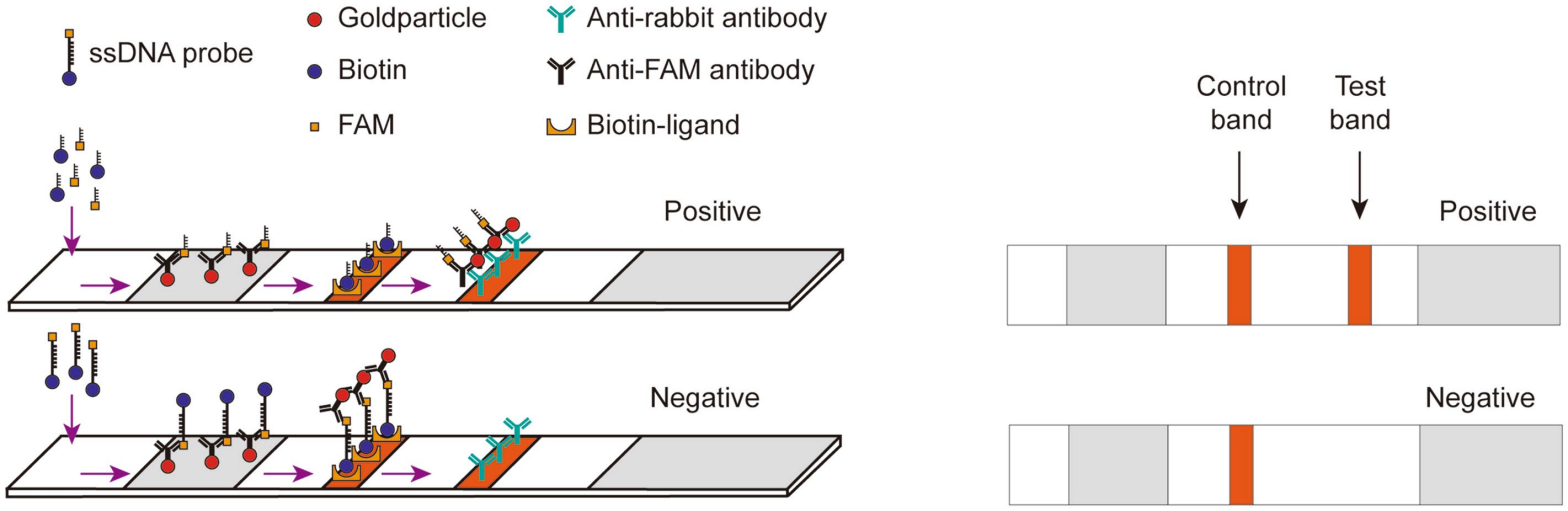
Schematic diagram of Lateral flow dipstick detection. Gold nanoparticles, conjugated with an anti-FAM antibody, were positioned on the binding pad. Streptavidin and IgG were fixed onto the NC membrane, serving as a control line for specific biotin binding and a test line for specifically binding to an anti-FAM antibody, respectively. The FAM-Biotin ssDNA reporter selectively adhered to the gold nanoparticles to create a complex due to the recognition of FAM by the anti-FAM antibody on the gold nanoparticles. When the Cas12a did not degrade the ssDNA, this complex bound to streptavidin at the control line. Conversely, when the ssDNA was degraded, leading to the release of Biotin from the complex, the complex was able to transit past the control line and bind to the IgG antibody at the test line.

The lateral flow detection technique could be described as follows (Figure 3): gold nanoparticles, conjugated with an anti-FAM antibody, were positioned on the binding pad. Streptavidin and IgG were fixed onto the NC membrane, serving as a control line for specific biotin binding and a test line for specifically binding to an anti-FAM antibody, respectively. The FAM-Biotin ssDNA reporter selectively adhered to the gold nanoparticles to create a complex due to the recognition of FAM by the anti-FAM antibody on the gold nanoparticles. When the Cas12a did not degrade the ssDNA, this complex bound to streptavidin at the control line. Conversely, when the ssDNA was degraded, leading to the release of Biotin from the complex, the complex was able to transit past the control line and bind to the IgG antibody at the test line.

The RPA reaction was conducted using the kit (TwistDx, United Kingdom), with the pUC57-NS1 pLasmid serving as the template. In a nutshell, the RPA amplification reaction mixture consisted of 2.4 μL of each RPA primers (10 μM), 29.5 μL of Rehydration Buffer, 2.5 μL of MgOAc (208 mM), 12.2 μL of ddH2O, and 1 μl of DNA. The total volume of the mixture was 50 μL, which was then incubated at 39°C for 20 min. Subsequently, a 1:1 mixture of 50 μL phenol/chloroform was added, and the blend was thoroughly mixed by agitation. After centrifugation at 12000 rpm for 5 min, 5–8 μL of the supernatant was analyzed via agarose gel electrophoresis for verification.

In each CRISPR/Cas12a-based fluorescence detection assay, the FAM-BHQ ssDNA receptor (5’-FAM-TTATT-BHQ-3′) was utilized. The reaction mixture consisted of 200 nM of LbCas12a (Bio-lifesci, Guangzhou, China), 100 nM of crRNA, 0.5 μL of RNase inhibitor, 3 μL of 10 × LbCas12a Buffer, 200 nM of FAM-BHQ1-labeled ssDNA reporter, and 3 μL of RPA product. Following thorough mixing in a 1.5 mL tube using a vortex, the mixture with a total volume of 25 μL was then incubated at 45°C for 20 min. Subsequently, the reaction was either photographed using a smartphone under blue light or imaged using a Gel Imaging System (Bio-Rad).

In the CRISPR/Cas12a-based lateral-flow strip assay for PPV7 detection, the FAM-biotin ssDNA probe labeled with FAM at the 5′ end and biotin at the 3′ end (5’-FAM-TTATT-Biotin-3′) was utilized. When PPV7 DNA is present in the reaction, both the C line and T line on the lateral flow strip will be visible. In contrast, if the sample is strongly positive, only the T line will be visible. The reaction was conducted using pUC57-NS1 plasmids as the template, along with a Cas-12a-based mixture reagent comprising 200 nM of Lba Cas12a, 100 nM crRNA, 200 nM FAM-Biotin ssDNA reporter, 0.5 μL of RNase inhibitor, 3 μL of LbCas12a buffer, 3 μL of RPA product, and 0.5 μL of RNase inhibitor, with the total volume made up to 50 μL using RNA-free water. The reaction was carried out at 45°C for 20 min. Following the reaction, the lateral flow strip (Bio-lifesci, Guangzhou, China) was inserted into the mixture, and the results were observed visually with the naked eyes within 10 min.

### 2.4 crRNA selection and concentration optimization

To select the most optimal crRNA for the RPA-Cas12a-based assay, reactions were carried out as described above using each of the 5 crRNAs. The copy number of the dsDNA target plasmid was tested at 105 copies/μl. To further optimize the crRNA concentration in the RPA-Cas12a-based assay, additional reactions were conducted with the same setup, but with varying crRNA concentrations. Specifically, the crRNA concentrations were adjusted to 200 nM, 400 nM, 1.2 μM, 2 μM, and 4 μM, respectively.

### 2.5 Concentration optimization of ssDNA reporter

For optimizing the ssDNA reporter concentration, reactions were carried out as above with the following modifications: the crRNA was set at the concentration optimized above. For each CRISPR/Cas12a-Based Fluorescence Detection Assay, the concentration of the FAM-BHQ ssDNA receptor was varied at 25 nM, 50 nM, 100 nM, 200 nM, 250 nM and 500 nM, respectively. Similarly, for each CRISPR/Cas12a based lateral-flow strip Assays, the concentration of the FAM-Biotin ssDNA reporter was adjusted to 50 nM, 100 nM, 200 nM, 500 nM and 1 μM, respectively.

### 2.6 Sensitivity and specificity evaluation of the RPA-Cas12a based assays

To assess the sensitivity of the developed RPA-Cas12a based assay, a concentration gradient of pUC57-NS1 plasmids ranging from 10^8^ to 10^0^ copies/μl was utilized as templates for the reactions. The assay’s limit of detection was determined as the minimum number of target plasmid copies required to produce a fluorescence signal or a visible test band. To validate the specificity of the developed RPA-Cas12a based assays, nucleic acid samples from five different porcine viruses were tested. These included viral genomic of PCV2, PCV3, PRV, cDNA from PRRSV and a mixture of viral genomic DNA from PPV2-PPV6. These nucleic acid samples were prepared and stored in our laboratory for testing purposes. Additionally, the sensitivity and specificity of the traditional PCR detection method were assessed using the same concentration gradient of pUC57-NS1 plasmids and the nucleic acid samples employed in the sensitivity and specificity evaluation of the RPA-Cas12a-based assays.

### 2.7 Detection of genomic DNA of clinical samples by RPA-Cas12a based assay

A total of 50 lung tissue samples obtained from ill pigs in various commercial pig farms in eastern Inner Mongolia were homogenized to extract DNA. All the pigs suffered from different clinical symptoms, mainly gastrointestinal or respiratory disease. Total genomic DNA of the samples was extracted by utilizing the TIANamp Virus DNA Kit (TIANGEN BIOTECH, Beijing, China) as previously described (33). Subsequently, all the DNA samples were tested for the presence of PPV7 using either the traditional PCR assay or the RPA-Cas12a based assays developed in this study.

### 2.8 Statistical analysis

Data were analyzed using GraphPad Prism software, version 10.1.2. Two-tailed Student t-test was used to analyzed the fluorescence difference between different reactions detected by CRISPR-Cas12a-based assay. *p* < 0.05 was considered statistically significant. Additionally, statistical analysis of the clinical detection results was performed using IBM SPSS Statistics 22 software through χ^2^ testing and Kappa consistency analysis, where a *p* value of <0.05 indicated a statistically significant difference.

## 3 Results

### 3.1 Selection and concentration optimization of crRNAs for Cas12a-based PPV7 detection

**Figure 4:**
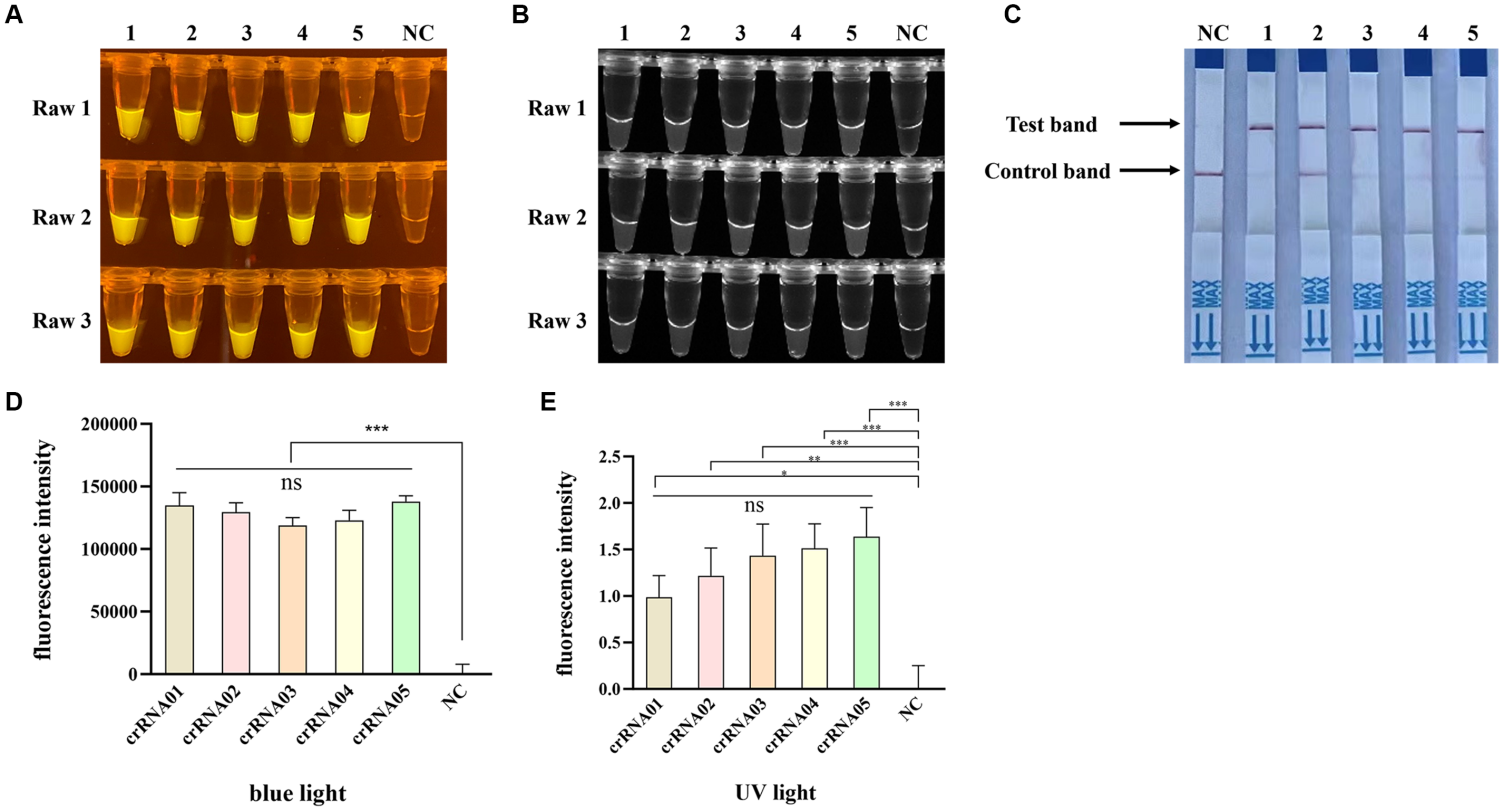
Selection of the optimal crRNA for PPV7 detection by CRISPR/Cas12a-based assay. The CRISPR/Cas12a based fluorescence detection assay using 5 different crRNAs under blue light **(A)** and UV light **(B)**. Lateral flow strip assay using 5 crRNAs **(C)**. Fluorescent intensity was calculated by Image J software **(D,E)**. DNA template represents conserved sequence of NS1 in pMD18T-NS1 plasmid; no. 1–5 represent crRNA-01-crRNA-05, respectively. NC: negative control (ddH_2_O). All the experiments were performed three time. Values are presented as means ± s.d. (error bars) (*n* = 3 replicates; * *p* < 0.05, ** *p* < 0.01, *** *p* < 0.001 between samples, two-sample *t*-test).

To obtain an optimal crRNA for Cas12a-based PPV7 detection, a total of 5 crRNAs (designated as crRNA-1 to crRNA-5) were synthesized and tested using the pUC57-NS1 plasmid containing the target region of the PPV7 NS1 gene as a template. Each crRNA was individually employed in Cas12a-based detection assays for PPV7. The results indicated that all crRNAs produced observable fluorescence signals under blue light (Figure 4A), and UV light (Figure 4B), and showed specific test bands (Figure 4C). Moreover, the fluorescent intensity of the reactions using crRNA1-5 under blue light (Figure 4D) or UV light (Figure 4E) calculated with Image J software were significantly higher than that of the negative control. However, there was no significant difference in fluorescence intensity or test band intensity among the reactions using the different crRNAs. Based on these findings, crRNA-05 was arbitrarily chosen for subsequent experiments.

**Figure 5:**
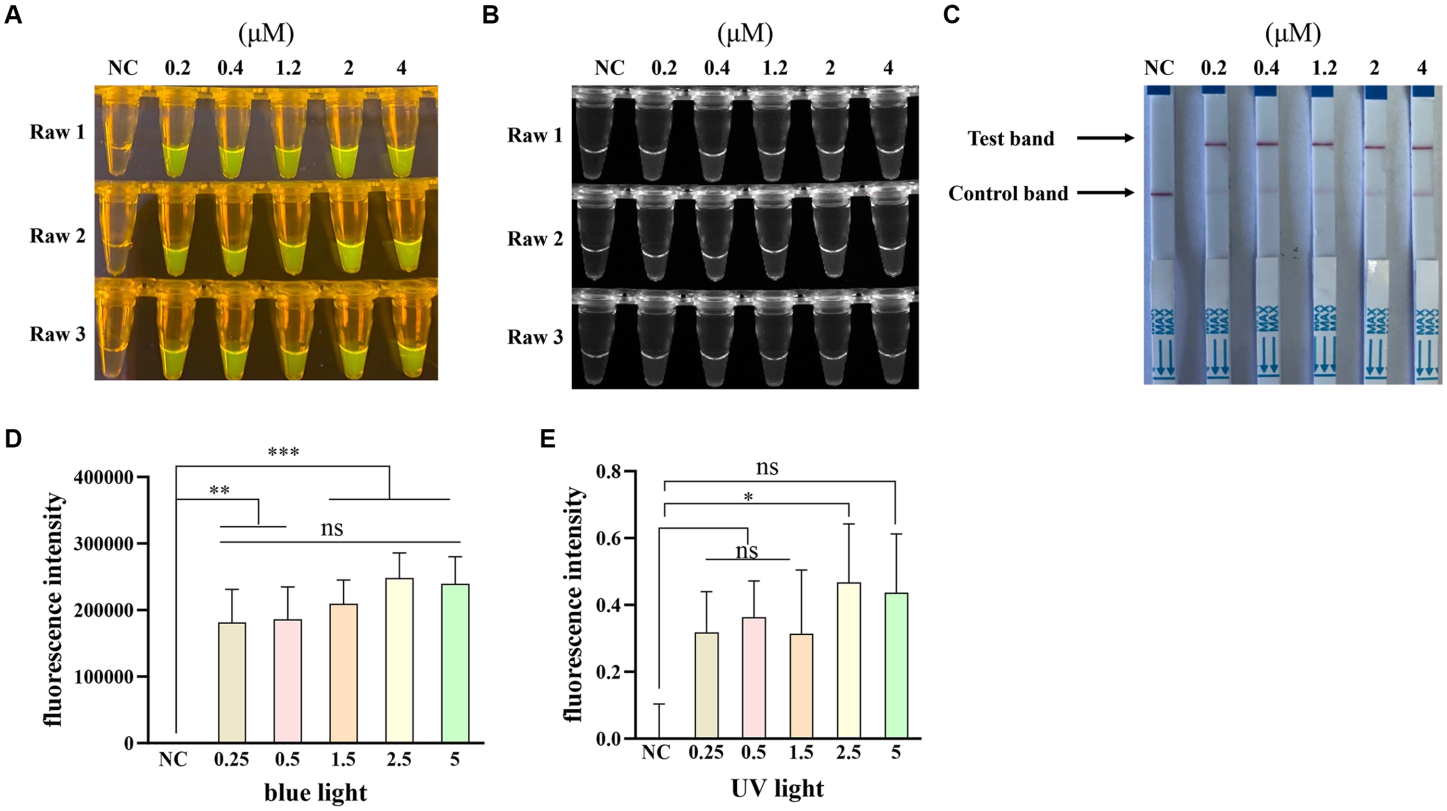
Optimization of the crRNA concentration. The CRISPR/Cas12a based fluorescence detection assay with different concentrations of crRNA under blue light **(A)** and UV light **(B)**. Lateral flow strip assay with different concentrations of crRNA **(C)**. Fluorescent intensity was calculated by Image J software **(D,E)**. NC: negative control (ddH_2_O). All the experiments were performed three time. Values are presented as means ± s.d. (error bars) (*n* = 3 replicates; * *p* < 0.05, ** *p* < 0.01, *** *p* < 0.001 between samples, two-sample *t*-test).

Furthermore, to optimize the crRNA concentration for the Cas12a-based PPV7 detection, five different concentrations (0.2 μM, 0.4 μM, 1.2 μM, 2 μM and 4 μM) of crRNA were tested in the reactions. The results showed that all concentrations led to the observation of fluorescence signals (Figures 5A,B) and specific test bands (Figure 5C) in the reactions. However, similar to the previous observations with different crRNAs, there was no significant difference in fluorescence intensity or test band intensity among the reactions using varying crRNA concentrations (Figures 5D,E). Given the lack of substantial differences in performance based on crRNA concentration, the lowest concentration of 200 nM was selected for further experiments.

### 3.2 Optimization of ssDNA concentration for Cas12a-based PPV7 detection

**Figure 6:**
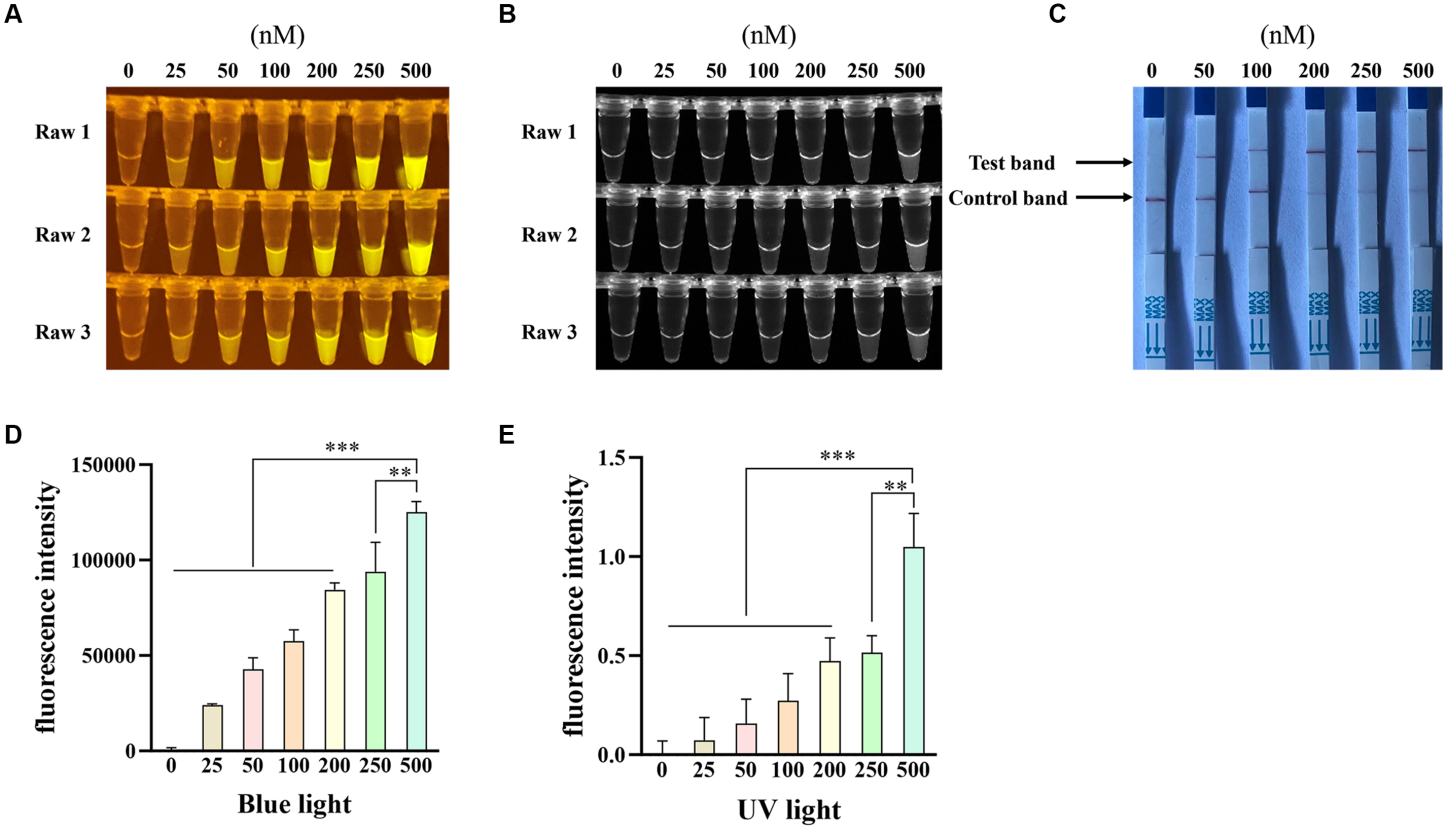
Optimization of the concentration of single-stranded DNA-fluorescent quencher (ssDNA-FQ) reporters and biotin for Porcine Parvovirus 7 (PPV7) detection by a CRISPR/Cas12a-based assay. The CRISPR/Cas12a based fluorescence detection assay with varying concentrations of ssDNA-FQ reporters under blue light **(A)** and UV light **(B)**. Lateral flow strip assay conducted with different concentrations of ssDNA-biotin **(C)**. NC: negative control (ddH_2_O). All the experiments were performed three time. Values are presented as means ± s.d. (error bars) (*n* = 3 replicates; * *p* < 0.05, ** *p* < 0.01, *** *p* < 0.001 between samples, two-sample *t*-test). Fluorescent intensity was calculated by Image J software (**D** and **E**).

To optimize the single-stranded DNA (ssDNA) concentration, we tested six different concentrations (25 nM, 50 nM, 100 nM, 200 nM, 250 nM, and 500 nM) of the FAM-BHQ ssDNA receptor and five different concentrations (50 nM, 100 nM, 200 nM, 250 nM, and 500 nM) of the FAM-biotin ssDNA reporter for the RAA-Cas12a assay. Figures 6A,B demonstrate that the reaction using 500 nM of the FAM-BHQ ssDNA receptor exhibited the strongest fluorescence. Additionally, the fluorescence intensity under blue light (Figure 6D) and UV light (Figure 6E) of the reaction using 500 nM ssDNA reporter was significantly higher than that of the other reactions, as calculated by Image J software. However, the most prominent test band was observed in reactions utilizing 200 nM, 250 nM, and 500 nM of the FAM-biotin ssDNA reporter (Figure 6C). Therefore, we selected a concentration of 500 nM for the FAM-BHQ ssDNA receptor and 200 nM for the FAM-biotin ssDNA reporter for subsequent experiments.

### 3.3 Sensitivity and specificity evaluation of the RPA-Cas12a based assays

**Figure 7:**
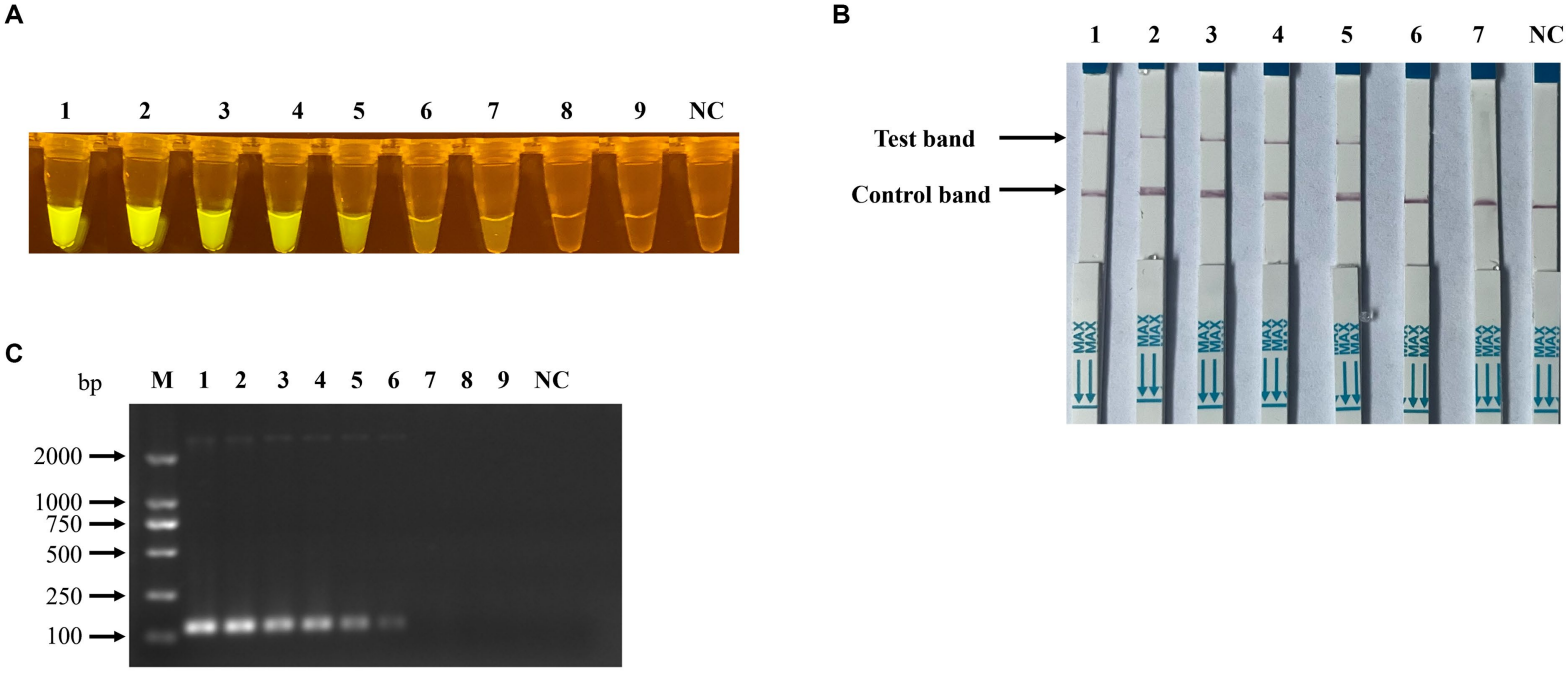
Sensitivity of RPA-CRISPR/Cas12a-based detection for PPV7. Serial 10-fold dilutions of PPV7 pUC57-NS1 plasmids (10^8^–10^0^ copies) were detected using the RPA-CRISPR/Cas12a-based fluorescence detection assay **(A)**, or (10^6^–10^0^ copies) were detected using the lateral-flow strip assay **(B)**. Additionally, the sensitivity of the traditional PCR detection method used in this study was evaluated using the same concentration gradient of pUC57-NS1 **(C)**. No. 1–9 represents plasmids with concentrations of 10^8^–10^0^ copies/μl in **(A,C)**, and no. 1–7 represents plasmids with concentrations of 10^6^–10^0^ copies/μl in **(B)**. NC: negative control (ddH_2_O).

**Figure 8:**
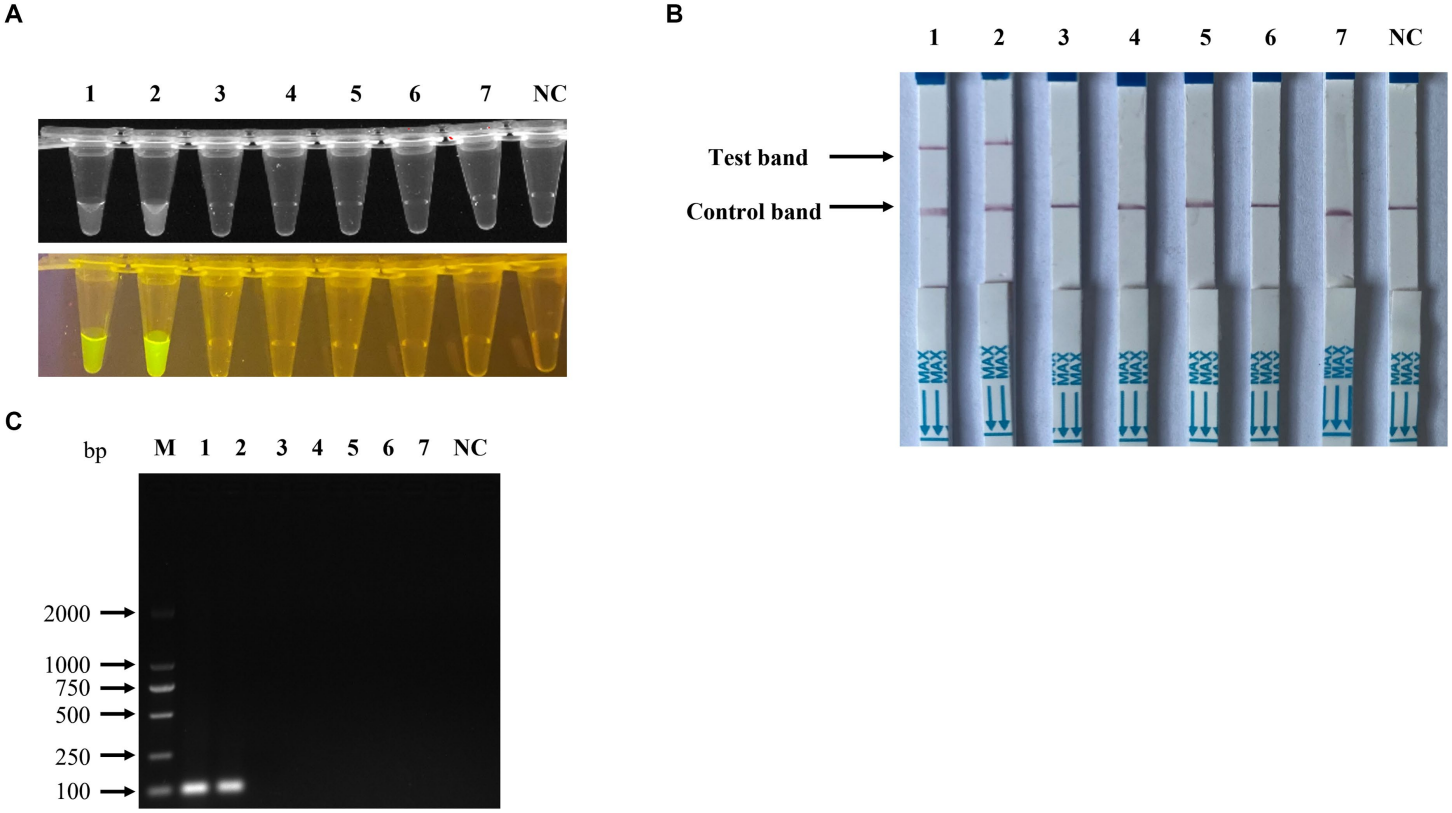
Specificity of the RPA-CRISPR/Cas12a-based detection for PPV7. The specificity analysis of the RPA-CRISPR/Cas12a-based fluorescence detection assay was carried out using the viral genomic DNA of a PPV2-PPV6 mixture, PRV, PCV2, PCV3, and complementary DNA (cDNA) of PRRSV in the fluorescence detection assay **(A)**, in the lateral-flow strip assay **(B)** and in the traditional PCR detection method **(C)**. No. 1–2 represents PPV7 pUC57-NS1 plasmids, while 3–7 represent the viral genomic DNA of the PPV2-PPV6 mixture, PRV, PCV2, PCV3, and cDNA of PRRSV. NC: negative control (ddH2O).

To assess the detection sensitivity of the recombinase polymerase amplification (RPA) combined with CRISPR/Cas12a-based assay, the pUC57-NS1 plasmid DNA was serially diluted ten-fold from 1 × 10^8^ to 1 × 10^0^ copies/μl and utilized as templates. The results indicated that the sensitivity of the RPA combined with CRISPR/Cas12a-based fluorescence detection (Figure 7A) and lateral-flow strip detection (Figure 7B) for Porcine Parvovirus 7 (PPV7) reached as low as 1 × 10^2^ copies/μl, which is 10 times more sensitive than the traditional PCR detection method (1 × 10^3^ copies/μl, Figure 7C). In the specificity analysis of the RPA-Cas12a-based PPV7 assays, only the PPV7 viral DNA sample exhibited positive results (Figures 8A,B). Similarly, in the specificity analysis of the traditional PCR detection assay, only the PPV7 viral DNA sample yielded positive results (Figure 8C).

### 3.4 Detection of PPV7 in clinical samples by the RPA-Cas12a based assays

To validate the precision of the RPA combined with Cas12a assays for clinical sample detection, a total of 50 lung tissue samples obtained from diseased pigs were utilized as test samples. The genomic DNA extracted from these samples was initially screened and confirmed using conventional PCR assays, followed by detection using RPA-CRISPR/Cas12a-based assays. The results revealed that 22 out of 50 (44%) samples tested positive with traditional PCR, while 29 out of 50 (58%) samples tested positive with RPA-CRISPR/Cas12a-based assays.

The detection results from the two methods were analyzed using a contingency table for χ^2^ testing (Table 2) and Kappa consistency analysis. The findings indicated a statistically significant difference between the two detection methods (χ^2^ = 7, *p* = 0.016). The Kappa consistency analysis yielded *p* = 0.000 and a Kappa value of 0.725, indicating a good level of agreement between the two methods.

**Table 2:** Clinical validation of RPA-CRISPR/Cas12a based detection assay.

RPA-CRISPR/Cas12a based detection assay	Traditional PCR detection		In total
	Positive samples	Negative samples	
Positive samples	22	7	29
Negative samples	0	21	21
In total	22	28	50

## 4 Discussion

Porcine Parvovirus 7 (PPV7) was first identified in the USA in 2016 as a novel and infectious strain of PPV, raising concerns about its potential impact on the swine industry. The virus has been detected in various samples, including serum, fecal swabs, nasal swabs, and lung tissues, hinting at various modes of transmission. Recent research conducted in eastern Inner Mongolia by our team revealed a notable prevalence of PPV7 in diseased pig lung tissues in multiple commercial pig farms, emphasizing its widespread distribution in the region (17). Due to its association with exacerbating infections such as PCV2, PCV3, and PRRSV, PPV7 poses a significant threat to the well-being of the pig industry, highlighting the necessity for effective detection methods to curb its transmission.

The single-stranded genomic DNA of PPV7 encodes two primary proteins: the non-structural protein 1 (NS1), crucial for viral replication, and the Cap protein, a key antigenic component of PPV7. While both the NS1 and Cap genes have been utilized as targets for PPV7 detection in various studies (7, 10, 12, 17, 34, 35), the NS1 gene is generally favored due to its higher conservation compared to the Cap gene sequence. Several methods for detecting PPV7 nucleic acids have been described in various studies. Kim et al. developed a multiplex PCR technique capable of detecting PPV1-PPV7, with a sensitivity of 3 × 10^3^ viral copies/μl specifically for PPV7 (35). Li et al. introduced a SYBR Green I-based real-time PCR assay that could detect as less as 35.6 copies/μl DNA template (36). These nucleic acid-based diagnostic methods, such as PCR and RT-PCR, exhibit high specificity and sensitivity in identifying PPV7. However, their reliance on costly equipment renders them unsuitable for resource-constrained field laboratories, highlighting the urgent need for a nucleic acid-based assay for PPV7 detection that is easily implementable with minimal instrumentation.

The CRISPR system, serving as a bacterial defense mechanism against foreign nucleic acids, harbors various Cas proteins with nuclease activity. Notably, Cas12a stands out for its DNA-targeting nuclease function, making it a valuable tool for gene editing through cleavage and collateral cleavage capabilities (37). Cas12a has shown promise in detecting pathogenic microorganisms (38), as demonstrated in studies utilizing a combination of recombinase polymerase amplification (RPA) and Cas12a assays for monkeypox virus and SARS-CoV-2 detection (39, 40). Unlike other Cas proteins like Cas13a and Cas13b, which focus on RNA sequences, Cas12a’s specificity lies in recognizing and cleaving DNA, thereby saving time by eliminating transcription steps in the construction of nucleic acid detection systems (41). Additionally, the absence of transcription reagents in the Cas12a reaction system simplifies the components, potentially enhancing accuracy. However, compared to other pathogens, there is currently a gap in developing CRISPR-based diagnostic methods for PPVs.

In this study, RPA primers were designed based on the NS1 sequences of PPV7 isolates available in GenBank, specifically targeting the most conserved regions. In addition to the RPA primers, the design of crRNAs is essential for the Cas12a-based detection system. Cas12a proteins require crRNAs to bind and initiate *trans*-cleavage of nonspecific ssDNA, recognizing sequences adjacent to the protospacer adjacent motif (PAM) sequence (TTTN or AAAN) within the target DNA template (23, 26, 42, 43). There are five PAM sites (TTTN or AAAN) in the RPA amplicons of PPV7, and corresponding sequences flanking these PAM sites were selected as crRNAs. These crRNA sequences were validated for specificity through Blast analysis against GenBank, confirming their specificity to the PPV7 NS1 protein gene.

Our study presented a rapid and easily deployable detection method requiring minimal equipment for monitoring PPV7 in pig farms. We meticulously optimized the reaction conditions, and the Cas12a-based assay demonstrated high sensitivity with a detection limit of 100 copies/μl, comparable to the SYBR Green I-based real-time PCR assay conducted by Li et al. (36). Furthermore, specificity evaluation and clinical sample testing revealed that the Cas12a-based diagnostic approach exhibited no cross-reactivity with other swine pathogens. In the clinical sample detection assay, 7 out of 50 samples exhibited inconsistent detection results, which were attributed to low viral loads that traditional PCR methods could not detect. The RPA-CRISPR/Cas12a-based assays demonstrated an improved detection rate for these low-concentration samples. Overall, the results suggest that the RPA-CRISPR/Cas12a-based assay for detecting PPV7 is more sensitive than traditional PCR methods, making it a reliable option for detecting samples collected from various pig farms.

## Data Availability

The original contributions presented in the study are included in the article, further inquiries can be directed to the corresponding authors.
